# Suicide Risk and Resilience in Stock Market Investors and Traders: Clinical and Medico-Legal Considerations

**DOI:** 10.3390/bs16050689

**Published:** 2026-04-30

**Authors:** Leo Sher

**Affiliations:** 1 Department of Mental Health, U.S. Department of Veterans Affairs James J. Peters Medical Center, 130 West Kingsbridge Road, New York, NY 10468, USA; leo.sher@mssm.edu; Tel.: +1-718-584-9000; Fax: +1-718-741-4703; 2 Department of Psychiatry, Icahn School of Medicine at Mount Sinai, New York, NY 10029, USA; 3 Department of Psychiatry, Columbia University Vagelos College of Physicians and Surgeons, New York, NY 10032, USA

**Keywords:** stock market, investor, trader, suicide, resilience, malpractice

## Abstract

Stock market investors and traders operate in high-pressure environments marked by volatility, uncertainty, financial risk, and intense performance demands. These conditions lead to substantial psychological distress, increasing vulnerability to psychiatric disorders and suicidal behavior. Key psychological risk factors in this population include acute financial loss, chronic stress, impulsivity, perfectionism, and identity fusion with professional performance. Evidence from behavioral psychology and clinical psychiatry indicates elevated rates of mood disorders, anxiety, and burnout in trading environments. Resilience—including emotional regulation, effective stress-coping mechanisms, strong social support, and cognitive flexibility—emerges as a critical protective factor that mitigates suicide risk and promotes adaptive functioning. Strengthening psychological resilience and implementing evidence-based mental-health strategies may help reduce suicide risk and support overall well-being. The medico-legal dimensions of this issue encompass duty of care within high-stress financial workplaces, clinical obligations related to suicide risk assessment and documentation, confidentiality and safety considerations, and questions of foreseeability of suicide in cases involving severe or catastrophic financial loss. Despite growing awareness of mental health challenges in financial professions, the intersection of suicide risk, resilience, and medico-legal responsibilities in this population remains underexplored. Further research is needed to refine assessment frameworks and develop targeted suicide prevention interventions for this at-risk group.

## 1. Introduction

Individuals working in high-stress professions—such as physicians, nurses, firefighters, law-enforcement officers, emergency medical personnel, and stock market traders—may experience elevated risks of suicidal ideation, suicide attempts, and suicide mortality compared with the general population (Boxer et al., 1995; Schernhammer & Colditz, 2004; Braquehais et al., 2016; Dutheil et al., 2019; Carson et al., 2023). For example, physicians experience a higher incidence of suicide than the general population, with meta-analytic data indicating an overall elevated relative risk and particularly higher rates among female physicians, whose suicide risk is estimated to be 2–4 times that of women in the general population (Dutheil et al., 2019). In the United States, it is commonly estimated that approximately 300–400 physicians die by suicide each year, corresponding to suicide rates of about 28–40 per 100,000 among male physicians, exceeding those observed in the general male population (Schernhammer & Colditz, 2004).

Chronic occupational stress, repeated exposure to trauma, long and irregular work hours, and burnout are key contributors to increased vulnerability to suicide among individuals in stress-related professions (Dutheil et al., 2019). Structural and cultural barriers within these professions, including stigma surrounding mental health care and concerns about confidentiality or professional repercussions, may further impede help-seeking behaviors.

This narrative review examines available evidence concerning suicide risk and resilience among stock market investors and traders. The objectives are to (1) summarize relevant research on financial stress and suicidality, (2) discuss psychological and occupational factors that may influence vulnerability, (3) consider how digital trading environments may shape risk, and (4) explore clinical and medico-legal considerations related to suicide prevention in financial contexts. We begin by outlining key definitions.

## 2. Definitions

*The stock market* is a public platform where investors can buy, sell, and trade shares of publicly listed companies, allowing businesses to raise capital while providing individuals with investment opportunities (Malkiel & Ellis, 2012). The stock market operates through exchanges, which facilitate transactions between buyers and sellers, with prices determined by supply and demand dynamics, company performance, and broader economic indicators (Bodie et al., 2022). Stock markets play crucial roles in global finance by ensuring liquidity and transparency in trading activities (Fabozzi, 2015; Shiller, 2015).

*Stock market traders* are individuals or institutions that buy and sell financial securities, such as stocks, bonds, and derivatives, in financial markets with the goal of generating profits from price movements (Fabozzi, 2015; Bodie et al., 2022). They analyze market data, company information, and economic indicators to make informed trading decisions over short- or long-term horizons. By providing liquidity and facilitating price discovery, stock market traders play a vital role in the efficient functioning of financial markets (Fabozzi, 2015).

*Stock market investors* are individuals or institutions that allocate capital to publicly traded companies by purchasing shares with the expectation of earning returns through dividends, capital appreciation, or both (Malkiel, 2019; Bodie et al., 2022). Unlike traders, investors typically focus on longer time horizons and base their decisions on fundamental analysis, including a company’s financial performance, competitive position, and growth prospects. Some investors also engage in trading; however, investors and traders are generally distinct groups. Nevertheless, both groups may encounter similar sources of stress.

*Resilience* refers to the capacity to adapt successfully in the face of adversity, stress, or significant life challenges (Masten, 2001; Luthar et al., 2000; Sher, 2019). It encompasses dynamic processes that enable individuals to maintain or regain psychological well-being despite exposure to risk or disruption. Empirical research highlights the roles of cognitive flexibility, emotional regulation, and supportive relationships in fostering resilient outcomes.

*Suicide* is the act of intentionally causing one’s own death, typically resulting from a complex interaction of psychological, biological, social, and environmental factors (Mann, 2003; Fazel & Runeson, 2020; Sher, 2019; Serafini et al., 2023; Sher & Oquendo, 2023). Suicidal behavior is a broader construct that includes suicidal ideation (thoughts about ending one’s life), suicide plans, suicide attempts, and suicide death. These behaviors often emerge in the context of mental health conditions such as depression, substance use disorders, and trauma-related disorders, but they may also be influenced by acute stressors, social isolation, and access to lethal means (Fazel & Runeson, 2020; Nock et al., 2008; Sher, 2019; Sher & Oquendo, 2023).

*Suicide-related medical malpractice* refers to a legal and ethical concept in which a healthcare professional or institution may be held liable for a patient’s suicide when it is alleged that the death resulted from a breach of the accepted standard of care (Simon, 2002; Sher, 2015; Berman, 2018). Central to this concept is the duty of care owed to patients, particularly when suicide risk is foreseeable based on clinical assessment, documented history, or expressed ideation.

Beyond medical contexts, the concept of *duty of care* has been extended to non-medical settings where institutions or professionals exercise control or responsibility over individuals at risk. In environments such as schools, workplaces, or other settings, liability may arise from failures in supervision, risk recognition, environmental safety, or timely response to warning signs (Gutheil & Appelbaum, 2000; Mishna et al., 2015). Although the legal standards in these settings differ from those applied to healthcare providers, the underlying ethical concern remains consistent: whether reasonable duties were fulfilled when suicide risk was foreseeable and potentially mitigable. This broader application highlights the importance of systemic and organizational responsibility in suicide prevention while acknowledging the inherent limits of predictability and control.

## 3. Search and Selection Approach

This narrative review was conducted to synthesize existing knowledge on suicide risk and resilience among stock market investors and traders. A targeted literature search was performed in PubMed, PsycINFO, Scopus, and Google Scholar for publications between 1980 and 2025 using combinations of keywords including “suicide,” “suicidal behavior,” “stock market,” “investors,” “traders,” “financial loss,” “economic stress,” and “resilience.” Priority was given to peer-reviewed empirical studies, systematic reviews, and meta-analyses examining suicide risk, occupational stress, financial stressors, or resilience. Because direct empirical research on traders and investors remains limited, additional literature from behavioral finance, occupational mental health, and suicidology was included to provide conceptual context. Non-peer-reviewed sources such as media reports were used only as illustrative examples and are explicitly identified as non-systematic cases.

## 4. Observations and Studies of Suicide Among Investors and Traders

### 4.1. Historical Perspective

The Bankers’ Panic of 1907 destabilized financial markets, causing U.S. stock prices to fall to roughly half of their previous year’s peak. Mortality statistics for 1908 reflect this turmoil, indicating that the proportion of deaths attributed to suicide among bankers, brokers, and company officials was more than twice that observed in the general population (U.S. Department of Commerce and Labor, 1909; Wisniewski et al., 2020).

Accounts of traders jumping from windows following the 1929 stock market crash circulated widely at the time (Rogers, 1929; McClain, 2018), although later commentators questioned their accuracy (Lowenthal, 1987; Galbraith, 1997). On 24 October 1929, the United States stock market experienced a dramatic collapse, prompting vivid contemporary descriptions. Will Rogers, a newspaper columnist, remarked, “When Wall Street took that tailspin, you had to stand in line to get a window to jump out of, and spectators were selling space for bodies in the East River” (Rogers, 1929). Reports of ruined stockbrokers leaping from tall buildings circulated widely at the time, though their factual accuracy remains contested. Suicides on Wall Street have been prominently depicted in the popular press and cinema (Stack & Bowman, 2014).

### 4.2. Illustrative Cases

Multiple cases of suicide among stock market investors and traders have been reported in both scholarly and lay literature. Several such cases are described in the following two paragraphs.

One of the most widely reported cases of suicide linked to stock trading involved a 20-year-old college student and retail trader who died by suicide after mistakenly believing he owed several hundred thousand dollars on a trading platform due to complex options trades (Klebnikov & Gara, 2020; Reuters, 2021). Historical accounts also cite the suicide of Jesse Lauriston Livermore, a prominent early 20th-century American stock trader who, after a series of financial successes and setbacks, died by suicide in 1940 amid personal and financial pressures (Millman, 1999).

In 2009, a 24-year-old equity sales trader who worked at Deutsche Bank died after jumping from the rooftop garden of the City restaurant in London, UK (Wilkinson, 2009). In 2019, a Bitcoin trader in China died by suicide after losing 2000 Bitcoins on a 100× leveraged position after entering a short trade that was liquidated when the market moved in the opposite direction (Emem, 2019). In November 2025, a chemical engineer in Uttarakhand, India, died by suicide after incurring substantial losses in the stock market (Press Trust of India, 2025). He reportedly created smoke by burning coal on a heater in his room and locked himself inside, leading to death by asphyxiation.

The incidents described in the preceding two paragraphs illustrate how acute financial stress, misunderstandings of trading risk, and perceived loss of control may intersect with mental health vulnerabilities among investors and traders. Although systematic research on suicide among traders and investors remains limited, such documented cases underscore the potential psychological impact of high-risk financial activities and the importance of mental health resources and risk education for individuals involved in trading. These cases are illustrative and do not establish prevalence or causal inference.

### 4.3. Research Studies

Kim et al. (2022) examined the effects of catastrophic financial loss on suicide risk using the stock market crash of October 2008 in South Korea as a natural experiment. The authors report that more than 11% of people aged 30–60 years were directly investing in stocks during the market crash. In October 2008, both major South Korean investor trading board indices dropped by 22.67% and 30.14%, respectively. The authors note that direct trading by individual investors is very common in the Korean stock market and that these investors are likely to hold less diversified stock portfolios. Thus, a substantial proportion of South Korean investors likely experienced extreme financial losses during the October 2008 stock market crash. In November 2008, the suicide rate among males aged 30–60 years increased by more than 40% compared with the expected levels if there had been no market crash. Among females aged 30–40 and 40–50 years, the suicide rate increased by 101.84% and 74.81%, respectively. The duration of the impact of the stock market crash on suicide differed between males and females. For males, the effect persisted throughout the nine-year sampling period. In females, the deleterious effect had a half-life of approximately 1.5 years and four months in the 30–40 and 40–50 age groups, respectively.

Gao et al. (2024) conducted an individual-level time-stratified case-crossover study to explore the association between daily stock volatility (daily returns and intra-daily oscillations for three types of stock indices) and major adverse cardiovascular events (MACEs) and suicide among more than 12 million individuals in mainland China between 2013 and 2019. For daily stock returns, both increases and decreases were associated with elevated mortality risks for all MACEs and suicide. There were also consistent and positive associations between intra-daily stock oscillations and mortality due to MACEs and suicide.

Another study reported that a low stock price index, daily declines in the stock index, and consecutive daily declines were associated with an increased risk of hospitalization following suicide attempts (Lin et al., 2017). Specifically, the stock price index had a significant impact on attempted suicide among individuals aged 45–54 years of both genders. Daily index changes were significant for both genders in the 25–34 and 55–64 age groups, whereas accumulated declines were significant only among females aged 25–44 and those aged 65 years and older.

Although the studies by Kim et al. (2022), Gao et al. (2024), and Lin et al. (2017) are based on general population data, stock market fluctuations primarily affect stock investors and traders. Studies by Kim et al. (2022) and Gao et al. (2024) explicitly acknowledge this focus by discussing the impact of market changes on investors. Notably, the term “investor” (or “investors”) appears 33 times in Kim et al. (2022) and six times in Gao et al. (2024), underscoring the centrality of investors to their analyses. Lin et al. (2017) note that shrinking wealth resulting from a declining stock market can lead to stress and depression, thereby implicating investors and traders as particularly vulnerable. It is important to emphasize that these studies examine associations between macroeconomic indicators and population-level health outcomes. Such analyses cannot determine whether individuals who died by suicide were themselves investors or traders or directly exposed to financial losses. Consequently, caution is required to avoid ecological fallacy—that is, inferring individual-level causal relationships from aggregate data. Future research incorporating individual exposure measures and financial-behavior data would be necessary to establish more direct causal relationships.

Despite the observations noted above, no rigorous research has examined whether stock market collapses directly contribute to suicide. In a society where success is often measured in material terms, it is reasonable to hypothesize that substantial financial losses could, in some cases, precipitate such desperate actions.

## 5. Factors Contributing to Suicidality Among Investors and Traders

**Figure 1:**
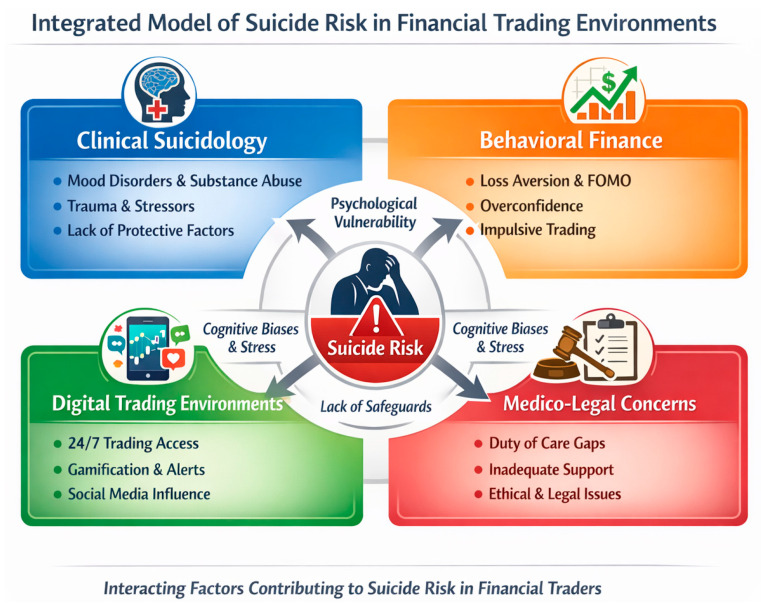
Integrated model of suicide risk in financial trading environments.

Suicidal behavior among stock market traders and investors often stems from acute financial stress and economic loss, which have been linked to increased suicide risk in empirical studies (Lin et al., 2017; Kim et al., 2022; Gao et al., 2024) (Figure 1). For example, population-level research shows that extreme stock market fluctuations and crashes are associated with higher suicide rates following sharp index declines, suggesting that catastrophic losses may have measurable impacts on mental health outcomes (Lin et al., 2017; Kim et al., 2022; Gao et al., 2024). Moreover, broader socioeconomic research has established that financial strain—defined by debt, unemployment, and lower income—is a significant predictor of suicide attempts and ideation, even after accounting for other risk factors (Ettman et al., 2020; Elbogen et al., 2020). These findings highlight how monetary loss and economic insecurity may translate into psychological distress that elevates suicide risk.

In addition to market losses, occupational stress and psychological vulnerability contribute to suicide risk among traders (Oberlechner & Nimgade, 2005; Abdulrahman et al., 2022). High job demands, performance pressure, and long hours characteristic of financial trading environments are associated with elevated stress and suicidality, consistent with meta-analytic evidence linking job stressors to suicidal thoughts and behaviors (Milner et al., 2018). High-frequency and cryptocurrency trading platforms present distinct risks compared with traditional investing. Real-time stock trading and cryptocurrency trading are associated with excessive gambling, gaming, and internet use behaviors, as well as higher psychological distress, perceived stress, and loneliness (Kasula & Alshboul, 2025; Oksanen et al., 2022).

Certain personality features, such as impulsivity, perfectionism, and loss aversion, may exacerbate emotional reactions to losses, while co-occurring mental health conditions such as depression and anxiety further increase vulnerability to suicide (Sher et al., 2001; Mann, 2003; Fazel & Runeson, 2020; Sher & Oquendo, 2023; Flett & Hewitt, 2024). Studies report elevated rates of anxiety, mood disturbances, emotional dysregulation, and burnout-related symptoms in trading and high-pressure financial settings (Abdulrahman et al., 2022; Fenton-O’Creevy et al., 2011; Lo et al., 2005). The most common comorbid mental health conditions among stock traders and investors at risk for suicidal behavior include mood disorders, substance use disorders, anxiety disorders, posttraumatic stress disorder (PTSD), and impulse-control disorders (Kessler et al., 2005; Nock et al., 2009). These conditions frequently co-occur. Social factors, including stigma surrounding financial failure and reduced help-seeking, may compound these effects, making it more difficult for at-risk traders and investors to access support when needed.

## 6. Suicide Risk Among Investors and Traders: Resilience, Institutional Responsibility and Duty of Care

Suicide among investors and traders represents a complex phenomenon shaped by the interplay of individual vulnerabilities and systemic pressures. Resilience provides a valuable framework for understanding why individuals exposed to similar financial stressors may experience markedly different psychological outcomes (Southwick et al., 2014). Psychological resilience encompasses the capacity for emotional regulation, tolerance of uncertainty, and cognitive flexibility in the face of losses (Connor & Davidson, 2003; Sher, 2019). Behavioral resilience is reflected in disciplined risk-management strategies, including position sizing, leverage control, and structured decision-making that mitigate impulsive responses to market fluctuations (Southwick et al., 2014). Social resilience, characterized by access to supportive interpersonal relationships and the willingness to seek help without fear of stigma, may further buffer against psychological distress (Kleiman & Liu, 2013). Existential resilience, or the maintenance of meaning and self-worth independent of financial outcomes, provides an additional protective layer against adversity (Southwick et al., 2014). Together, these dimensions may attenuate the psychological impact of financial losses and reduce vulnerability to suicidal behavior (Connor & Davidson, 2003; Kleiman & Liu, 2013; Sher, 2019).

Beyond individual-level factors, the concept of duty of care provides a complementary lens for examining institutional and systemic responsibilities (Shneidman, 1996; Jobes & Joiner, 2019). Traditionally applied within healthcare settings, suicide-related malpractice refers to failures in duty of care when suicide risk is foreseeable and potentially preventable (Shneidman, 1996). When extended to financial contexts, this concept raises ethical questions regarding the design and operation of trading platforms and financial products. Features such as high leverage availability, gamification, constant performance feedback, and limited safeguards following extreme losses may inadvertently exacerbate psychological distress (Hollis & Kelly, 2020). The absence of cooling-off mechanisms, proactive outreach, or meaningful warnings during periods of severe loss may further contribute to harm among vulnerable individuals (Luxton et al., 2012).

The increasing reliance on online trading platforms and algorithmic financial systems may also influence how the concepts of foreseeability and duty of care are interpreted in financial contexts. Unlike traditional trading floors, where traders interacted with colleagues, supervisors, and brokers who could observe behavioral changes and potentially intervene during periods of distress, contemporary digital trading environments often involve minimal direct human interaction. Retail investors may engage with markets primarily through automated interfaces, mobile applications, and algorithm-driven information displays. This shift can reduce opportunities for social monitoring, informal peer support, and early recognition of psychological distress. At the same time, digital platforms control the presentation of complex financial data, leverage options, and risk indicators. Consequently, questions may arise regarding the foreseeability of harm when platform design, automated alerts, or account displays could potentially mislead inexperienced users or amplify emotional responses to losses. Although financial institutions are not responsible for providing clinical care, ethical and legal discussions increasingly emphasize the importance of transparent risk communication, clear presentation of account information, and responsible platform design as elements of organizational duty of care in digitally mediated financial environments (Černevičienė & Kabašinskas, 2024; Weber et al., 2024; Devarajulu et al., 2025).

It is essential to emphasize that the application of duty of care in this domain does not imply that financial institutions function as mental health providers. Rather, it underscores the ethical relevance of foreseeable psychological harm arising from business practices and technological design (Hollis & Kelly, 2020; Jobes & Joiner, 2019). A public health-oriented approach would prioritize harm reduction through mental health-informed platform design, transparent risk communication, optional self-limiting tools, and partnerships with mental health professionals (Luxton et al., 2012). Such measures may complement individual resilience-building efforts and contribute to suicide prevention at the population level (Shneidman, 1996; Jobes & Joiner, 2019).

## 7. How Can Suicides Among Traders and Investors Be Prevented?

**Figure 2:**
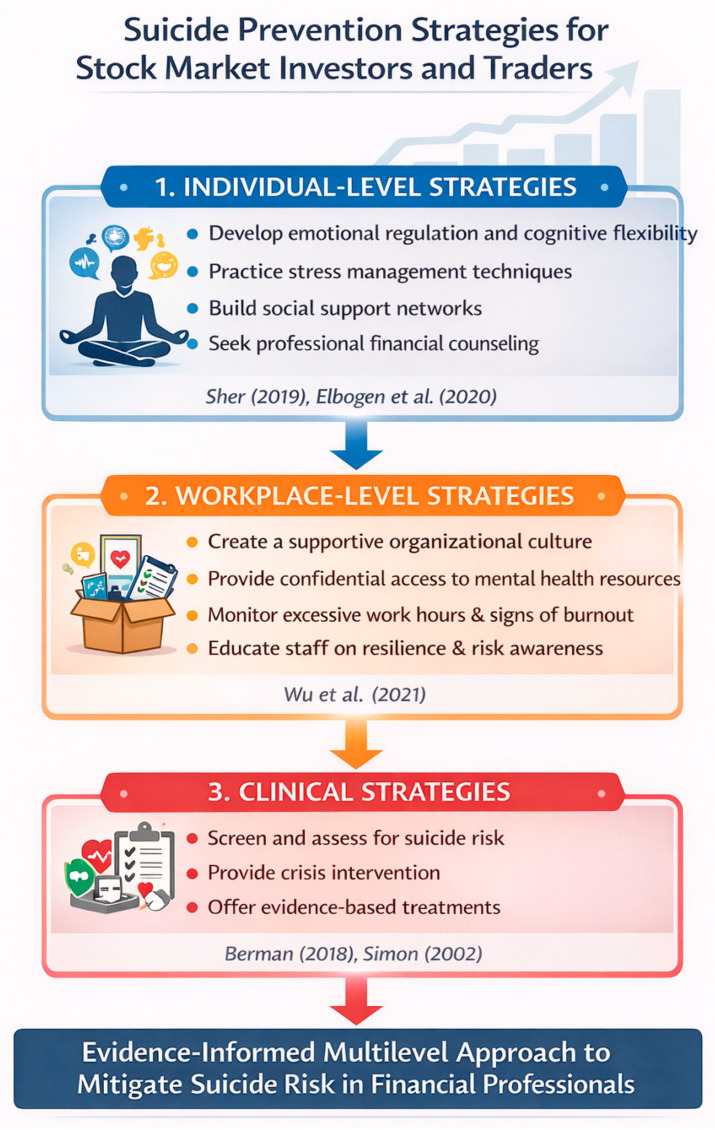
Suicide prevention strategies for stock market investors and traders (Sher, 2019; Elbogen et al., 2020; Wu et al., 2021; Berman, 2018; Simon, 2002).

Preventing suicides among traders and investors requires a holistic approach that addresses both psychological and financial stressors intensified by market volatility (Figure 2). Research shows that financial stress and market volatility can be associated with increased suicide risk during periods of economic turmoil, highlighting the need for proactive measures to support mental health in high-pressure financial environments (Agrrawal et al., 2017; Roelfs & Shor, 2023).

### 7.1. Individual-Level Strategies

In the trading context, emotional responses such as fear, stress, and physiological reactions influence decision-making and cognitive function, suggesting that traders’ emotional regulation and stress awareness are as important as financial expertise (Lo & Repin, 2001). Awareness and education are critical first steps: traders need to recognize early warning signs of distress, including anxiety, depression, insomnia, or impulsive decision-making.

A practical daily routine for traders and investors may begin with morning preparation, such as a few minutes of mindfulness, deep breathing, or meditation to center oneself and set realistic expectations for the day. During work, it may be helpful to balance focus with self-care by taking breaks, monitoring emotions, and recognizing signs of stress or frustration, while logging feelings to support self-awareness. After the trading day, individuals may consider reflection, journaling experiences, normalizing setbacks, celebrating small successes, engaging in physical activity or social connection, and seeking mental health support if needed. Following such practices regularly may help promote emotional well-being and resilience.

### 7.2. Workplace and Organizational Strategies

Organizations also play an important role in supporting mental health among traders and investors. Access to mental health services and resilience-building interventions is essential. Resilience has been recognized as a protective factor against suicidal risk and psychological distress (Sher, 2019; Wang et al., 2022; American Psychological Association, 2023). Studies indicate that higher resilience is associated with lower suicide risk and may moderate the relationship between stress and suicidal ideation (Sher, 2019; Elbogen et al., 2020; Edwards et al., 2025).

Organizations can foster resilience by creating supportive work environments, monitoring high-risk behavior, and implementing workplace mental health best practices such as psychosocial safety climates and access to counseling (Wu et al., 2021). Exchanges and brokerages can also encourage access to mental health resources, provide training on resilience and coping strategies, and monitor patterns of stress or burnout among traders.

### 7.3. Clinical and Public Health Strategies

Clinical and public health approaches may complement individual and organizational efforts. Early identification of mental health conditions, access to counseling and psychiatric care, and the availability of crisis support services are essential components of suicide prevention. Public health strategies that promote mental health awareness and reduce stigma surrounding help-seeking may further support individuals experiencing financial stress.

By integrating individual coping skills, organizational support, and broader clinical and public health strategies, the financial industry may help reduce suicide risk and cultivate resilience, enabling traders and investors to recover from losses and sustain long-term well-being.

## 8. Conclusions

Stock market investors and traders operate in high-pressure environments characterized by volatility, uncertainty, and financial risk, which contribute to psychological distress and heightened vulnerability to psychiatric disorders and suicidal behavior. Key risk factors include acute financial loss, chronic occupational stress, impulsivity, perfectionism, and identity fusion with performance outcomes. Empirical studies report elevated rates of mood disorders, anxiety, and burnout within trading populations. Psychological resilience—including emotional regulation, effective coping strategies, social support, and cognitive flexibility—serves as a critical protective factor. Medico-legal considerations encompass duty of care, suicide risk assessment, confidentiality, and foreseeability following substantial financial loss. Despite increasing recognition of mental health challenges in financial professions, the intersection of suicide risk, resilience, and medico-legal responsibilities in this population remains underexplored, underscoring the need for further research and targeted suicide-prevention strategies.

## Data Availability

Data sharing is not applicable.
